# The Selective Transport of Ions in Charged Nanopore with Combined Multi-Physics Fields

**DOI:** 10.3390/ma14227012

**Published:** 2021-11-19

**Authors:** Pengfei Ma, Jianxiang Zheng, Danting Zhao, Wenjie Zhang, Gonghao Lu, Lingxin Lin, Zeyuan Zhao, Zijing Huang, Liuxuan Cao

**Affiliations:** 1 College of Energy, Xiamen University, Xiamen 361005, China; 32420191152349@stu.xmu.edu.cn (P.M.); zwu@xmu.edu.cn (J.Z.); zhaodanting@stu.xmu.edu.cn (D.Z.); zhangwenjie@stu.xmu.edu.cn (W.Z.); 32420182202926@stu.xmu.edu.cn (G.L.); 32420181153423@stu.xmu.edu.cn (L.L.); 2 Fujian Research Center for Nuclear Engineering, Xiamen 361005, China; 3 Fujian Key Laboratory of Functional Marine Sensing Materials, Minjiang University, Fuzhou 350108, China; zyzhao@mju.edu.cn

**Keywords:** ion selectivity, multi-physics fields, charged nanopore, electric field, hydraulic pressure

## Abstract

The selective transport of ions in nanopores attracts broad interest due to their potential applications in chemical separation, ion filtration, seawater desalination, and energy conversion. The ion selectivity based on the ion dehydration and steric hindrance is still limited by the very similar diameter between different hydrated ions. The selectivity can only separate specific ion species, lacking a general separation effect. Herein, we report the highly ionic selective transport in charged nanopore through the combination of hydraulic pressure and electric field. Based on the coupled Poisson–Nernst–Planck (PNP) and Navier–Stokes (NS) equations, the calculation results suggest that the coupling of hydraulic pressure and electric field can significantly enhance the ion selectivity compared to the results under the single driven force of hydraulic pressure or electric field. Different from the material-property-based ion selective transport, this method endows the general separation effect between different kinds of ions. Through the appropriate combination of hydraulic pressure and electric field, an extremely high selectivity ratio can be achieved. Further in-depth analysis reveals the influence of nanopore diameter, surface charge density and ionic strength on the selectivity ratio. These findings provide a potential route for high-performance ionic selective transport and separation in nanofluidic systems.

## 1. Introduction

Nanoporous membranes attract broad interest due to their unique mass transport property and potential applications in DNA sequencing [1,2], molecular separation [3,4], chemical sensing [5,6], ion filtration [7], seawater desalination [8,9], and energy conversion [10,11]. The extraordinary property stems from the surface of the nanopore which provides exceptional interaction compared to the counterpart of the macro scale [12,13,14]. It leads to the selective transport of ions, which is similar to the biological ion channel across the cell membrane. This selective transport stems from the polarization effect near the pore entrance and the physical and chemical properties of the surface, resulting in anomalous ion selectivity, ionic conductance enhancement, and ionic current rectification [15,16,17].

To achieve the highly selective ionic transport, the material property has been widely explored as one of the crucial factors. Given the performance of state-of-the-art membranes is limited by the solution-diffusion mechanism, in which the ion or molecules dissolve in the material first and then diffuse across the membrane [18], the materials containing nanoscale confined the space of pores provide new solutions to this problem [19,20]. Nanopores with geometrical dimensions smaller than, or comparable to, the diameter of hydrated ions are expected to enhance ion selectivity [21,22]. This is because dehydration will occur while the ion is transported through such atomic-scale sieves. For instance, Geim’s group reported the graphene-based two-dimensional materials can achieve tunable ion sieving through the additional energy barriers induced by the capillary size [23,24]. Fang’s group reported the high ion selectivity through the ion intercalation to control interlayer spacing in graphene oxide membranes (GOM) [25]. Wang’s group prepared latent-track nanopores on polymer materials, which can achieve a high selectivity of lithium and magnesium through subnanometer pores [26]. Huanting Wang’s research group constructed polymer and metal-organic frameworks (MOF), which obtained a high selectivity of mono/divalent ions [27].

Despite extensive research on ion dehydration effects, the ion selective transport controlled by dehydration is still limited by the difficulties in fabricating membranes with well-defined uniform subnanometre pores [28]. Additionally, due to the very similar diameter between the different hydrated ions, the selectivity based on steric hindrance, can only separate specific ion species, lacking of universal separation effect. In this regard, several externally tunable approaches have been proposed so far. Most of them change ionic transport properties by regulating the nanochannel wall property triggered by external chemical stimuli, such as pH value [29], enzymes [30] and polyvalent cations [31]. Moreover, the ionic transport also was regulated by nanopore size [32], temperature [33], ionic type and strength [29]. Contrary to the chemical stimuli-responsive strategies, more physical fields or external driving forces are induced to control the ionic transport behaviors. The existing experimental literature suggested the different responses of individual ions. In particular, the transport rate of different monovalent ions is different under osmotic pressure [34,35]. Similarly, driven by the hydraulic pressure, the ionic currents between different ions are also distinct [36,37]. These experimental phenomena implied that the highly selective ion transport may be achieved under the synergy of multi-physics fields in the nanopores. However, related research is still absent.

Herein, we propose the highly ionic selective transport in charged nanopore through the coupled effect of hydraulic pressure and electric field. Based on the Poisson–Nernst–Planck (PNP) and Navier–Stokes (NS) equations, the ion transport behaviors in the confined space were analyzed under the framework of the continuity model [38,39,40]. The validity of the method was confirmed by previous literature [41,42]. The results show that the coupling of hydraulic pressure and electric field can significantly enhance the ion selectivity compared to the results under the single driven force of hydraulic pressure or electric field. Moreover, through the synergy of opposite hydraulic pressure and electric field, the transportation state of target ions can be precisely controlled. It accounts for the highly selective transport from other kinds of ions. Further in-depth theoretical analysis reveals the influence of nanopore diameter, surface charge density and ionic strength on the selectivity ratio. These findings provide a potential route for the general strategy of the ionic selective transport and the inspiration for the design of high-performance nanofluidic systems.

## 2. Materials and Methods

### 2.1. Numerical Calculation

The ion transport through the charged nanopore was analyzed under the framework of the continuum model based on the coupled Poisson–Nernst–Planck (PNP) and Navier–Stokes (NS) equations. Since the calculation results are consistent with the Brownian dynamics simulation, it shows that the continuity model is accurate enough when the pore size is greater than 2 nm [42,43]. The finite element method [44] was used to solve the coupled partial equations with the applied hydraulic pressure and electric potential and other boundary conditions, yielding the ionic densities and liquid flows in the systems. The net current contributed by each kind of ion can be obtained by integrating the ionic flow density over the cross section of the nanopore. The commercial calculation software COMSOL was employed to solve the coupled differential equations, which is widely used in the calculation of multi-physical fields. In particular, the calculation modules included transfer of dilute substances, electrostatic substance and laminar flow [45].

The Poisson equation controls the relationship between electric potential and ion concentrations [46]:

(1) $$\nabla^{2}\Phi =-\frac{1}{\varepsilon }\sum_{i}z_{i}ec_{i}\;\;\;i=+-$$

Here, *ε* stands for the dielectric constant of solution.

The transport characteristics of charged nanopore can be described by Nernst–Planck equation as follows for each ionic species:

(2) $$\vec{j_{i}}\;=-D_{i}\left(\nabla c_{i}+\frac{z_{i}{\mathrm{ec}}_{i}}{k_{B}T}\nabla \Phi \right)\;\;\;i=+,-$$

where *i* is the ion species, $\vec{j_{i}}$ stands for the ionic flux, $D_{i}$ stands for the diffusion coefficient, $c_{i}$ stands for the ion concentration, *z_i_* stands for the valence, Φ stands for the electrical potential, *T,* *k_B_,* *e* stand for the temperature, the Boltzmann constant, and the electron charge, respectively.

The Navier–Stokes (NS) equation describes how pressure and electrical potential drive the flow and determine the flow distribution, which can be expressed as [47,48]:

(3) $$\vec{u}\cdot \nabla \vec{u\;}=\frac{1}{\rho }\left[-\nabla p+\mu \nabla^{2}\vec{u}-\left(c_{+}-c_{-}\right)\nabla \Phi \right]=0\;\;i=+,-$$

where $\vec{u}$ stands for the fluid velocity, ρ stands for the mass density of solution, *p* stands for the pressure and μ is the viscosity of solution.

The steady-state solution should satisfy the continuity equation:

(4) $$\vec{\nabla }\cdot (c_{i}\cdot \vec{u}+\vec{j_{i}\;})=0\;\;\;i=+,-$$

For the incompressible flow, it should satisfy the condition:

(5) $$\vec{\nabla }\;\cdot \;\vec{u\;}=0\;\;\;i=+,-$$

The boundary condition of potential *Φ* is determined by Gauss law, which can be expressed as [49]:

(6) $$-\vec{n}\cdot \nabla \Phi =\frac{\sigma }{\varepsilon }\;\;\;\;i=+,-$$

where $\vec{n}$ is the unit vector in the normal direction; *σ* represents the charge density distributed on the inner wall of the nanopore.

In addition, the normal component of the local ion flux is 0 at the boundary:

(7) $$\vec{n}\cdot \vec{j_{i}\;}=0\;\;i=+,-$$

The detailed boundary conditions were summarized in Table S1 and Figure S1.

### 2.2. Models Parameters

**Figure 1:**
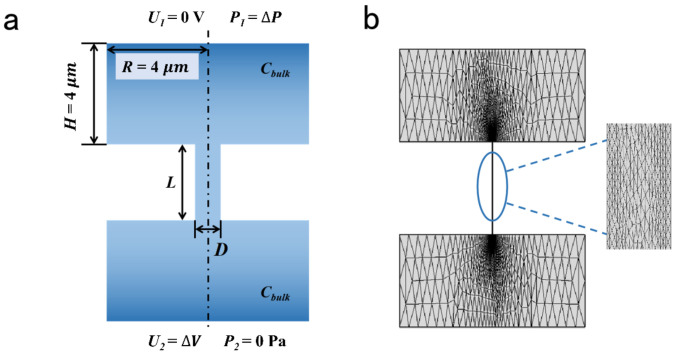
Calculation model and mesh analysis. (**a**) Schematic illustration of charged nanopore. (**b**) The grid in computational domain.

A 2D axisymmetric model was employed to calculate the ionic selective transport across the charged nanopore [38,39,50] (Figure 1). The two reservoirs were connected by the charged channels. The size of the reservoirs was 8 μm × 4 μm, which was large enough to figure out accurate results [51]. The voltage and pressure were applied as the boundary condition. The length of the charged pores was 1 μm; the pore diameter ranged from 4 nm to 20 nm; the charge density of the inner wall was set from −0.1 to −0.01 C/m^2^. The parameters above have been sufficiently discussed in many previous studies [42]. The detailed parameters of the calculation model are listed in Table 1.

## 3. Results and Discussion

The ion transport through the nanochannel was analyzed under the framework of the continuum model. In particular, the combined Poisson–Nernst–Planck (PNP) equations and the Navier–Stokes (NS) equations were used to calculate the ionic current in nanofluidic channels. The finite element method was used to solve the coupled partial equations, yielding the local ionic densities and ion flux in the system. The total ionic current through the nanopore could be calculated by integrating the ionic flow density over the cross section of the nanopore. When the ions transport within the negatively charged nanochannel, the cations were preferentially transported over anions owing to the Coulomb interaction. Under an external physical field, the preferentially selected cations exhibited different transport characteristics, which could be utilized to realize the separation of different ions.

**Figure 2:**
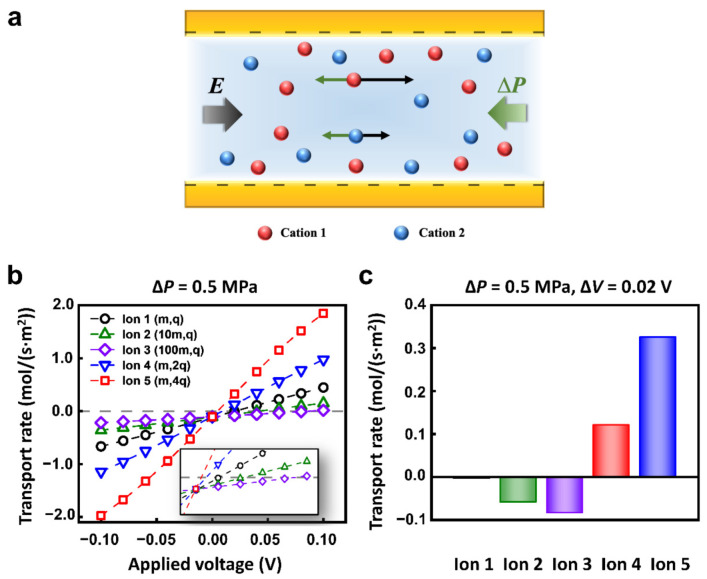
The selective transport of ions under multi-physical fields. (**a**) Schematic of the origin of selectivity. The ions with different charges and masses can achieve distinct motion state. (**b**) The different ions have a special response to the applied physical fields owing to the different masses and charges. (**c**) The selective transport of different ions under appropriate hydraulic pressure and electric potential conditions.

The ions with different charges and masses could achieve a distinct state of motion under the electric field and hydraulic pressure (Figure 2a). To quantitatively explore the transport behavior of different ions, we set 5 kinds of cations in the calculated models. The mass and charge number of Ion 1, 2, 3, 4, and 5 were (m, +q), (10m, +q), (100m, +q), (m, +2q), (m, +4q), respectively. The anions were all (m, −q). To simplify the calculation, different diffusion coefficients were used to represent ions of different quality [52]. The concentration of each kind of ion was 1 mM. The nanopore was 1 μm in length and 10 nm in diameter. The surface charge density was set to −0.06 C m^−2^, which was consistent with the value used in literature [53,54,55]. The nanopore was cation-selective. We mainly focused on the transport of cations to study the selective transport between cations.

When the opposite electric field and hydraulic pressure are applied across the nanopore, the *I–V* responses of Ion 1-5 are different. In particular, when the hydraulic pressure was fixed to 0.5 MPa, the calculated ionic currents increased monotonously with applied voltage (Figure 2b). However, the reversal electric potential conditions to keep the zero migration current of each ion are different. Therefore, the separation or selective transport of specific ions can be effectively achieved through the appropriate combination of electric field and hydraulic pressure. For example, with the applied voltage of 0.02 V and hydraulic pressure of 0.5 MPa, 5 kinds of ions exhibit different migration behaviors. The net flux of Ion 1 keeps a nearly zero flux state; Ion 2 and Ion 3 migrate in a negative direction; Ion 4 and Ion 5 transport in the positive direction (Figure 2c).

**Figure 3:**
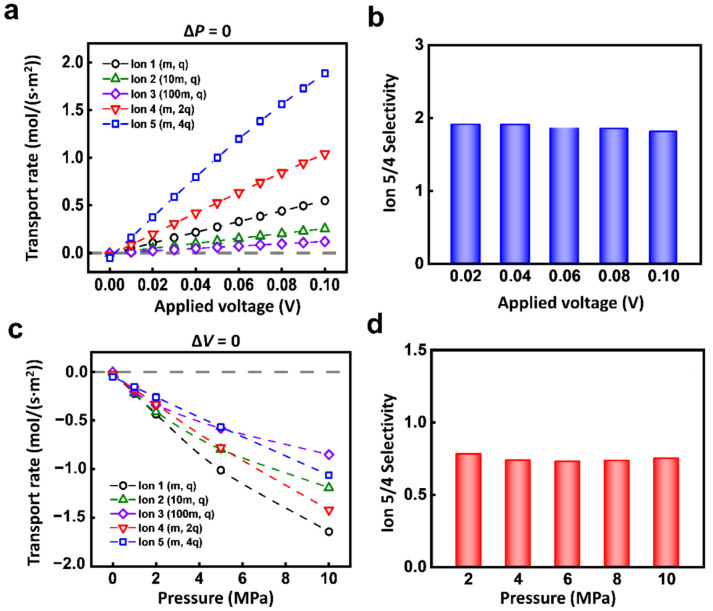
The ion transport under a single physical field. (**a**) Different charged ions have different responses to the electric field. (**b**) The selectivity ratio of Ion 4 and Ion 5 under electric field. (**c**) Different ions have different responses to hydraulic pressure. (**d**) The selectivity ratio of Ion 4 and Ion 5 under hydraulic pressure.

This high selectivity only occurs under the condition of coupled electric field and hydraulic pressure. If the ions are driven by a single physical field, the selectivity ratio of ion transport is relatively low. As shown in Figure 3a, under the electric field, the transport rates of the 5 ions all increase linearly with the voltage. The responses of Ion 1-5 are different. The larger the charge mass ratio is, the more significant the response to the electric field is. Due to the near-linear increment of different ions, the selectivity ratio between 2 kinds of ions is almost the same as the increment of the electric field. For instance, the selectivity ratio of Ion 5 to Ion 4 is stable at about 2 (Figure 3b).

The single hydraulic pressure also can lead to similar ion selectivity owing to the individual response of each kind of ion. The ionic transport rate monotonically increases with applied hydraulic pressure (Figure 3c). The selectivity ratio of Ion 5 to Ion 4 is also stable (Figure 3d). Among different kinds of ions, it is general that to achieve selectivity through a single physical field (Figure S2). Though the single physical field can generate the selective transport between different ions, the obtained selectivity ratio is extremely limited.

**Figure 4:**
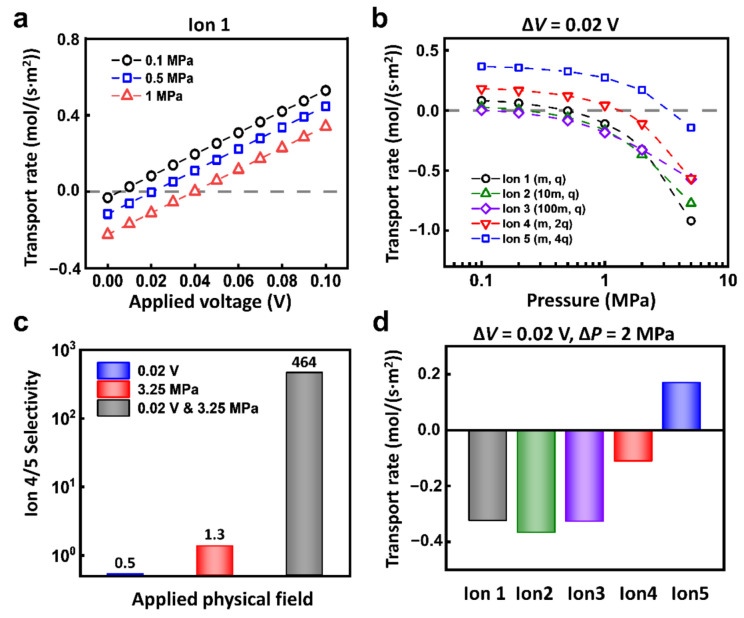
The multi-physical fields can evidently enhance ion selectivity. (**a**) Under fixed hydraulic pressure conditions, the ionic transport responds to the electric field. (**b**) Under the voltage condition of 0.02 V, the ionic transport of different ions can be regulated by applied hydraulic pressure. (**c**) The selectivity ratio of Ion 4 and Ion 5 under 0.02 V and 3.25 MPa is much higher than that under a single physical field. (**d**) Through the combination of appropriate electric potential and hydraulic pressure, the efficient separation of target ions (for instance, Ion 5) can be achieved.

Through the coupling of electric field and hydraulic pressure, the selectivity ratio of ion transport can be largely improved. Take Ion 1 for example, when the reverse hydraulic pressure is applied, the ions transport in the negative direction. With the enhancement of forward electric field, the ionic current is drawn back to zero and then grows along the direction of the applied electric field (Figure 4a). Under the different conditions of hydraulic pressure, the required reverse voltage to balance the hydraulic pressure is different as well (Figure S3). Such equilibrium conditions are different between different ions. As shown in Figure 4b, under the applied voltage of 0.02 V, the response of each kind of ion to the hydraulic pressure is different. For example, when the hydraulic pressure increases to 0.4 MPa, the driven force of hydraulic pressure is larger than the electric field force in Ion 2 and Ion 3; the electric field force is larger in Ion 4 and Ion 5; the two forces basically reach equilibrium in Ion 1. The selectivity ratios of different ions are closely related to the hydraulic pressure. With the increment of the applied hydraulic pressure, the selectivity ratios change greatly.

In this regard, through the appropriate combination of hydraulic pressure and electric field, we can realize high selectivity ratio of two kinds of ions. As shown in Figure 4c, to separate Ion 4 and 5, under the condition of 0.02 V electric potential and 3.25 MPa pressure, the separation ratio of Ion 4 and 5 can reach 464. In sharp contrast, the separation ratio under a single physics field is merely below 2. Furthermore, the coupled hydraulic pressure and electric field can also separate target ions from mixed ions. As shown in Figure 4d, under the condition of 0.02 V and 2 MPa, Ion 5 can pass through the nanochannel, while Ion 1, Ion 2, Ion 3, and Ion 4 are all completely rejected. 

**Figure 5:**
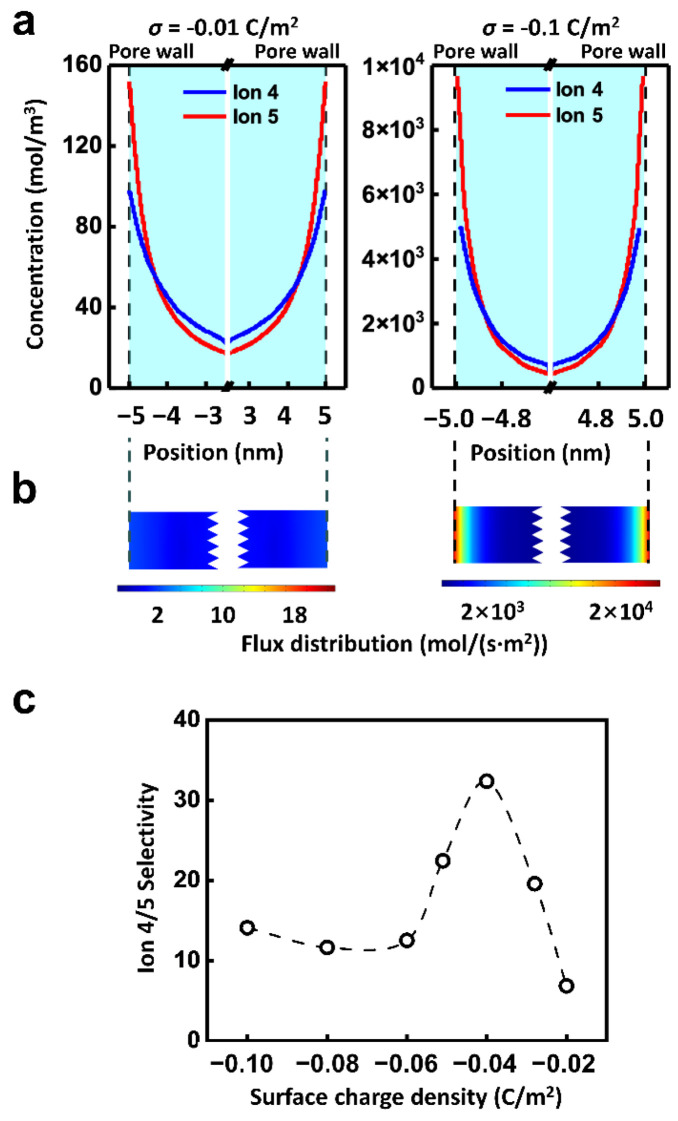
Effect of surface charge. (**a**) The radial concentration distribution of Ion 5 suggests that, the surface charge density largely affects the ion concentration distribution. The ion concentration near the pore wall is much higher than that in the center of the pore, indicating the importance of EDL. (**b**) The distribution of ionic flux in the channel. (**c**) The selectivity is regulated by the surface charge density. The well-matched multi-physics field condition and surface charge density can improve the selectivity ratio. The calculation condition is 0.01 V and 0.5 MPa.

Besides the applied physics fields, the surface charge of the channel is also a crucial influential factor of ionic selective transport. Because of the electrostatic interaction, the charge carried by the inner wall of the nanopores regulates the ion transport through the electric double layer (EDL) [56]. As shown in Figure 5a, the ion concentration within the nanochannel rises remarkably with the increment of the surface charge density. Along the radial direction, the ion strength goes up with the decrease of the distance from the inner wall. Thus, driven by the electric field, there is the high ion flux near the inner wall and the lowest ion flux in the center of the nanochannel. However, the ion migration properties driven by hydraulic pressure are quite different, which transports the most slowly at the wall and the fastest at the center of the channel (Figure S4). It is in accord with the reported literature [57].

Finally, the ionic flux of Ion 5 under the combined hydraulic pressure and electric field is shown in Figure 5b. It can be concluded that the total net ionic flux is dominated by the EDL. However, the two driven forces are quite different in the EDL region. The effect of electric field on ions is uniform in space distribution, while the effect of hydraulic pressure is stronger in the center of the nanochannel than that of the wall. It leads to the complexity of the ion transport and selectivity ratio under the combined hydraulic pressure and electric field. And the distribution of ion 4 is similar as shown in Figure S5. For instance, the effect of surface charge density on selectivity is not monotonic. As shown in Figure 5c, when the electric potential of 0.01 V and the pressure of 0.5 MPa are combined, the selectivity ratio of Ion 4 to Ion 5 grows with the increment of the surface charge density from 0.02 C/m^2^ to 0.04 C/m^2^. When the surface charge density enhances further, the selectivity ratio declines. The highest selectivity ratio can be achieved by matching the appropriate multi-physics field condition with the surface charge density. 

**Figure 6:**
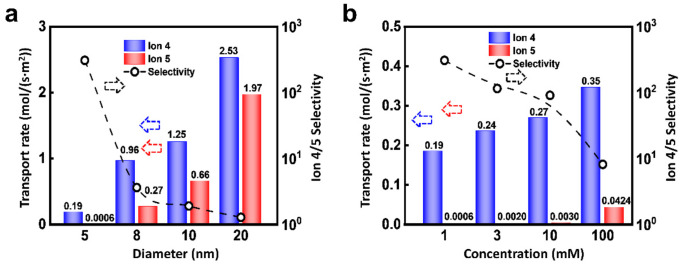
Effect of channel size and concentration. (**a**) Increasing channel radius can enhance the flux of ions, while it will lead to decreased selectivity; (**b**) An increase in ion concentration changes the thickness of the EDL, thereby affecting the migration of ions.

Due to the dominant role of EDL, the nanopore size can affect the ionic selected transport apparently. As shown in Figure 6a, under the condition of 0.02 V and 10 MPa, the flux of Ion 4 and 5 both increase with the pore size, but the selectivity ratio decreases. As the diameter of the nanochannel increases, the proportion of EDL is reduced, which accordingly leads to the drop of selectivity ratio. In addition, the selectivity ratio will also be regulated by the ion concentration. As shown in Figure 6b, the flux of Ion 4 is higher than that of Ion 5. Furthermore, the flux of Ion 5 is extremely small when the concentration is low. The fluxes of Ion 4 and 5 both increase with the concentration and the selectivity ratio decrease monotonously. This is because the increment of ion concentration reduces the thickness of EDL through the charge shielding effect [58]. Therefore, the relatively small-sized nanopore is beneficial to the highly selective ion transport. 

## 4. Conclusions

We systematically study the ion selective transport in charged nanochannels driven by the hydraulic pressure and electric field in theory. We can report the conclusions as follows:

1. The combination of electric field and hydraulic pressure can remarkably enhance the selectivity ratio of different ions. Through the synergy of opposite hydraulic pressure and electric field, the transportation state of target ions can be precisely controlled.
2. The EDLs play the predominant role in the ionic selective transport. The ion migration driven by the electric field and hydraulic pressure perform different behaviors in the EDL region. Through the optimized matching of EDL and applied multi-physics fields, the high selectivity ratio can be achieved.
3. Different from the material-based ion selectivity, in which the predefined ionic transport properties are impossible to change once the devices are fabricated, this approach can obtain a regulable selectivity towards the different ions.

The numerical calculations based on PNP-NS equations allow the quantitative physical description of the microscopic transport mechanism. These findings provide the necessary inspiration to the development of the high-performance ion separation systems in the experiment. The continuity model can not accurately describe the interaction on atomic and molecular scales. Therefore, these physical and chemical parameters are approximated in the calculation, including the coefficient of activity, the effective viscosity and the slip length on the wall. The more precise description needs more exploration in experiment and theory.

## Figures and Tables

**Table 1:** Parameters of calculation models.

Parameter	Description	Value	Parameters Involved in the Manuscript
*R*	Radius of the pool	4 μm	Figures 1–6, Figures S2–S5
*H*	Height of the pool	4 μm	Figures 1–6, Figures S2–S5
*L*	Length of nanopore	1 μm	Figures 1–6, Figures S2–S5
*D*	Diameter of nanopore	10 nm	Figures 1–5, Figures S2–S5
		5, 8, 10, 20 nm	Figure 6a
		5 nm	Figure 6b
*σ*	Charge density	−0.06 C/m^2^	Figures 1–4, 6, Figures S2–S4
		−0.02–−0.10 C/m^2^	Figure 5c
		−0.01, −0.10 C/m^2^	Figure 5a,b, Figure S5
*C*_bulk_	Ion concentration	1 mM	Figures 2–5, 6a
		1, 3, 10, 100 mM	Figure 6b
		1 mM	Figures S2–S5
∆*V*	Applied voltage	−0.1–0.1 V	Figure 2b
		0–0.1 V	Figures 3, 4a Figure S2a,b, Figure S3
		0.01 V	Figure 5, Figure S5
		0.02 V	Figure 2c, Figure 4b–d, Figure 6
∆*P*	Applied pressure	0.5 MPa	Figure 2, Figure 5, Figure S5
		0–10 MPa	Figure 3, Figure 4b, Figure S2c,d
		0.1, 0.5, 1 MPa	Figure 4a, Figure S3
		3.25 MPa	Figure 4c
		2 MPa	Figure 4d
		10 MPa	Figure 6

## Data Availability

The data presented in this study are available in Supplementary Material here.

## Supplementary Materials

The following are available online at https://www.mdpi.com/article/10.3390/ma14227012/s1, Supplementary Table S1: The boundary conditions, Supplementary Figure S1: The boundary condition label, Supplementary Figure S2: The ion selectivity under single physics field, Supplementary Figure S3: Different ionic transport responds, Supplementary Figure S4: The distribution of flow velocity, Supplementary Figure S5: The distribution of ionic flux of Ion 4.

## Nomenclature

GOM	graphene oxide membrane
MOF	metal-organic frameworks
PNP	Poisson–Nernst–Planck
NS	Navier–Stokes
EDL	electric double layer
*ϕ*	electrical potential
*ε*	dielectric constant
T	temperature
*p*	pressure
$\vec{n}$	unit vector
*σ*	charge density
*i*	ion specie
$\vec{j_{i}}$	ionic flux
$D_{i}$	diffusion coefficient
$c_{i}$	ion concentration
μ	viscosity
*z_i_*	valence
$\vec{u}$	fluid velocity
*k_B_*	Boltzmann constant
*e*	electron charge
ρ	mass density
