# Supplementary material for: The Selective Transport of Ions in Charged Nanopore with Combined Multi-Physics Fields
Source: Materials (Basel). 2021 Nov 19;14(22):7012. doi: 10.3390/ma14227012 (PMC8622219; doi:10.3390/ma14227012)
Supplement: Supplementary file 1 [file materials-14-07012-s001.zip › materials-1421331-SM.pdf]

Supplementary Materials

The Selective transport of ions in Charged Nanopore with Combined Multi-physics Fields

Pengfei Ma [1], Jianxiang Zheng [1,2], Danting Zhao [1], Wenjie Zhang [1], Gonghao Lu [1], Lingxin Lin [1], Zeyuan Zhao [3], Zijing Huang [1,2,*] and Liuxuan Cao [1,2,*]

[1] College of Energy, Xiamen University, Xiamen, Fujian 361005, China; 32420191152349@stu.xmu.edu.cn (P.M.); zwu@xmu.edu.cn (J.Z.); zhaodanting@stu.xmu.edu.cn (D.Z.); zhangwenjie@stu.xmu.edu.cn (W.Z.); 32420182202926.xmu.edu.stu.cn (G.L.); 32420181153423@stu.xmu.edu.cn (L.L.)

[2] Fujian Research Center for Unclear Engineering, Xiamen, Fujian 361005, China

[3] Fujian Key Laboratory of Functional Marine Sensing Materials, Minjiang University, Fuzhou, Fujian 350108, China; zyzhao@mju.edu.cn (Z.Z.)

* Correspondence: caoliuxuan@xmu.edu.cn (L.C.); fritz@xmu.edu.cn (Z.H.)

Table of the content:

1.Setting of boundary conditions.

2. The selectivity of single physics field.
3. Different ionic transport responses.
4. Distribution of fluid flux.
5. The flux of Ion 4.

1. Setting of boundary conditions.

Under the electrostatic field, the dependent variable is the electric potential V, and the corresponding governing equation is:

$$\nabla \cdot D = \rho_V \, , E = -\nabla V \tag{1}$$

Boundary condition 1: Conservation of charge:

$$\nabla \cdot (\varepsilon_0 \varepsilon_r E) = \rho_V \, , D = \varepsilon_0 \varepsilon_r E \tag{2}$$

Boundary condition 2: Relationship between surface charge density and the electric field:

$$n \cdot (D_1 - D_2) = \rho_s \tag{3}$$

Boundary condition 3: Zero potential, the wall of the solution pool is selected as zero potential:

$$V = 0 \tag{4}$$

The dependent variables in the transfer field of dilute substances are cation concentration c_p and anion concentration c_n. The transfer mechanism includes convection and ion migration in electric field:

$$\nabla \cdot J + u \cdot \nabla c_i = 0 \tag{5}$$

$$J = -D_i \nabla c_i - z_i u_{m,i} F c_i \nabla V \tag{6}$$

D_i represents the diffusion coefficient of the ion i, z_i indicates the charge number of the ion i, the mobility $u_{m,i}$ satisfies the Nernst-Einstein relationship:

$$u_{m,i} = -\frac{D_i}{RT} \tag{7}$$

The dependent variables of the laminar flow field are the velocity field u and the pressure

p:

$$\rho(u \cdot \nabla)u = \nabla[-pl + K] + F \tag{8}$$

$$\rho\nabla \cdot (u) = 0 \tag{9}$$

$$K = \mu(\nabla u + (\nabla u)^{\mathrm{T}}) \tag{10}$$

ρ represents the density of the fluid, μ represents dynamic viscosity coefficient of the fluid.

The corresponding boundary conditions are set as shown in the Figure S1 and Table S1 below.

Table S1. The boundary conditions

Boundary	Name	Equation
2, 3, 4, 5, 7, 8, 9	No Flux	$-\boldsymbol{n} \cdot (-\boldsymbol{D}\nabla \boldsymbol{c}) = 0$
1, 6	Concentration	$c = c_0$
2, 3, 4, 5, 7, 8	Zero Charge	$\boldsymbol{n} \cdot \boldsymbol{D} = 0$
9	Surface Charge Density	$\boldsymbol{n} \cdot (\boldsymbol{D1} - \boldsymbol{D2}) = \rho_S$
2, 3, 4, 5, 7, 8, 9	Wall	$\boldsymbol{u} = \boldsymbol{0}$
6	Electric Potential	$\boldsymbol{V} = \boldsymbol{0}$
	Pressure Condition	$\boldsymbol{P} = \boldsymbol{P_0}$
1	Electric Potential	$\boldsymbol{V} = \boldsymbol{V_0}$
	Pressure Condition	$\boldsymbol{P} = \boldsymbol{0}$

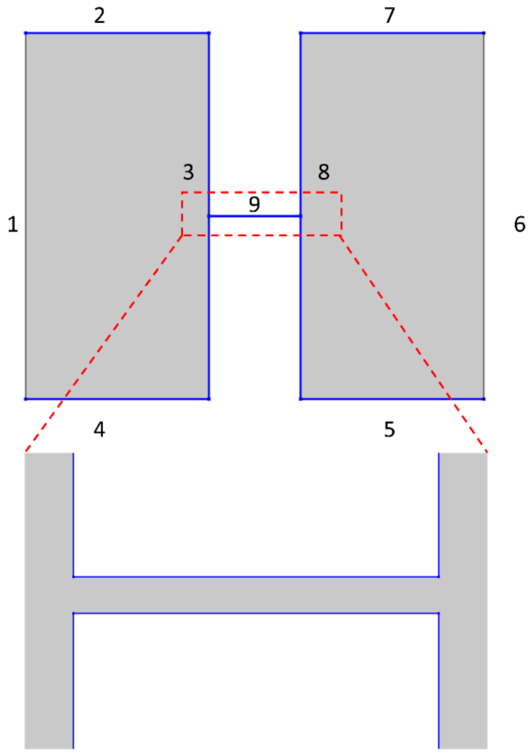

Figure S1. The boundary condition labels

2. The selectivity in single physics field.

Under the action of a single physical field, the ion selectivity is low. As shown in Figures S2a and S2b, the separation ratio is stable under different voltage conditions. For ion 1/2 and ion 1/4, the separation ratio is maintained at about 2 and 0.5, respectively. Under the hydraulic pressure, as shown in Figures S2c and S2d, the separation ratio is slightly affected by the magnitude of hydraulic pressure, but the influence is not significant. Similarly, the separation ratio is also below 2.

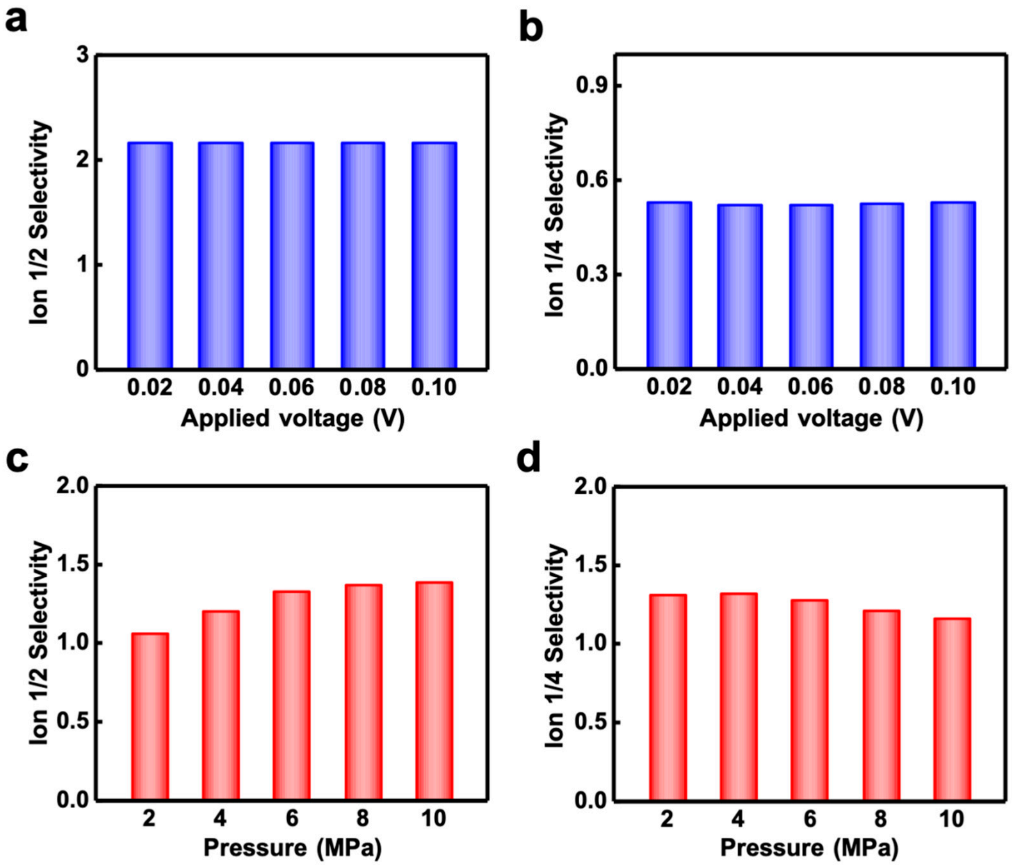

Figure S2. The ion selectivity under single physics field. (**a**) The selectivity of ion 1/2 is nearly 2 and maintain stable under different voltages. (**b**) The selectivity of ion 1/4 is nearly 0.5 and maintain stable under different voltages. (**c**) The selectivity of ion 1/2 is nearly 1.2 under different pressure. (**d**) The selectivity of ion 1/4 is nearly 1.2 under different pressure.

3. Different ionic transport responses.

For the same ion, the initial state is different under the applied pressure conditions. With the increment of applied pressure, the applied reverse voltage to make ion transport approaching zero is different. The responses of ion 2, 3, 4, and 5 are shown in Figure S3, and this difference exists for each ion.

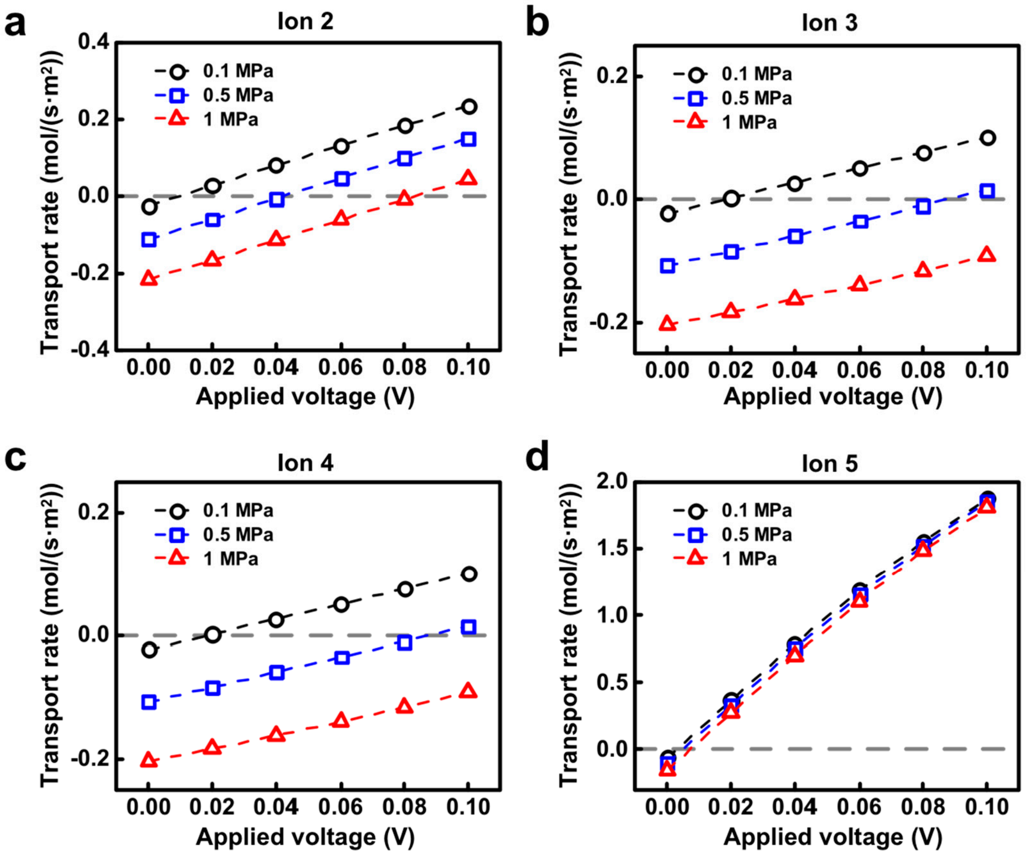

Figure S3. Different ionic transport response. Response of ion 2 (**a**), ion 3 (**b**), ion 4 (**c**) and ion 5 (**d**).

4. Distribution of fluid flux.

The distribution of flow velocity in the channel is shown in Figure S4. Because of the friction on nanopore wall, liquid flows slowest at the wall and fastest at the center of the channel. This migration properties driven by hydraulic pressure is quite different from that of electric field.

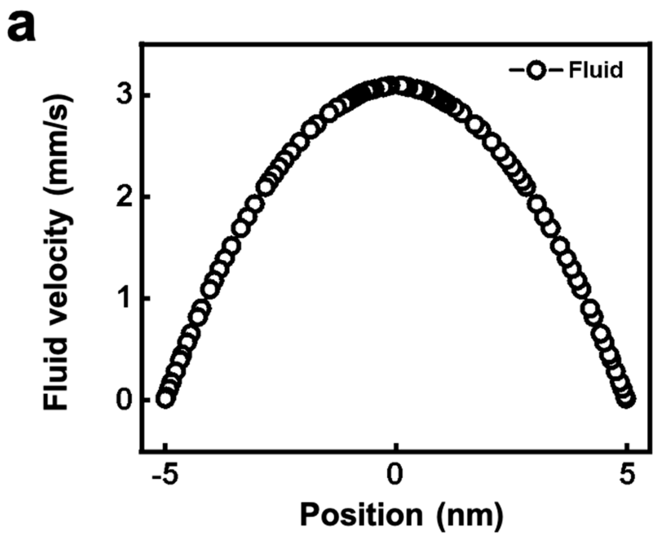

Figure S4. The distribution of flow velocity. The velocity of the fluid in the channel approaches zero at the wall and reaches the fastest at the center of the channel.

5. The flux of Ion 4.

Due to the total ionic flux is dominated by the EDL, the ionic distribution mainly concentrated on both sides of the wall. And the higher the surface charge is, the more obvious the effect is. These two driving forces are very different in the EDL area. The effect of the electric field on the ions is uniform in spatial distribution. But the hydraulic effect in the center of the nanochannel is stronger than the wall surface. This leads to the complexity of ion selective transport under the combined action of hydraulic pressure and electric field.

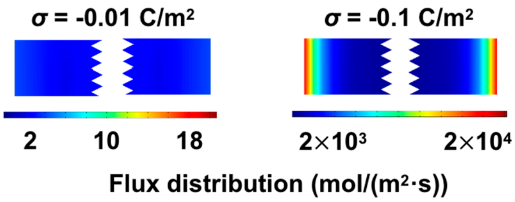

Figure S5. The distribution of ionic flux of Ion 4. The distribution of ionic flux in the channel is affected by the surface charge. The higher surface charge makes the higher ion concentration within EDLs.
